# Cholinergic Synapse Pathway Gene Polymorphisms Associated With Late-Phase Responses in Allergic Rhinitis

**DOI:** 10.3389/falgy.2021.724328

**Published:** 2021-08-16

**Authors:** Simranjit K. Samra, Ashwini Rajasekaran, Andrew J. Sandford, Anne K. Ellis, Scott J. Tebbutt

**Affiliations:** ^1^Experimental Medicine, University of British Columbia, Vancouver, BC, Canada; ^2^Centre for Heart Lung Innovation, St. Paul's Hospital, Vancouver, BC, Canada; ^3^Prevention of Organ Failure (PROOF) Centre of Excellence, Vancouver, BC, Canada; ^4^Department of Medicine, Division of Respiratory Medicine, University of British Columbia, Vancouver, BC, Canada; ^5^Departments of Medicine and Biomedical & Molecular Science, Queen's University, Kingston, ON, Canada; ^6^Allergy Research Unit, Kingston General Hospital, Kingston, ON, Canada

**Keywords:** environmental exposure unit, genetics, nasal congestion, allergic rhinitis, inflammation, late-phase response

## Abstract

Allergic rhinitis (AR) is characterized by an early-phase response (EPR), and in a subgroup of individuals, a late-phase response (LPR). We sought to investigate polymorphisms in cholinergic synapse pathway genes, previously associated with late-asthmatic responses, in the LPR. Twenty healthy participants and 74 participants with AR underwent allergen exposure using the Environmental Exposure Unit. Allergic participants were sub-phenotyped using self-reported nasal congestion scores; congestion is the predominant symptom experienced during the LPR. Acute congestion (AC, *n* = 36) participants developed only an EPR, while persistent congestion (PC, *n* = 38) participants developed both allergic responses. We interrogated blood samples collected before allergen exposure with genotyping and gene expression assays. Twenty-five SNPs located in *ADCY3, AKT3, CACNA1S, CHRM3, CHRNB2, GNG4*, and *KCNQ4* had significantly different allele frequencies (*P* < 0.10) between PC and AC participants. PC participants had increased minor allele content (*P* = 0.009) in the 25 SNPs compared to AC participants. Two SNPs in *AKT3* were associated with gene expression differences (FDR < 0.01) in PC participants. This study identified an association between the LPR and polymorphisms in the cholinergic synapse pathway genes, and developed a novel method to sub-phenotype AR using self-reported nasal congestion scores.

## Introduction

Allergic rhinitis (AR) is the most prevalent clinical manifestation of allergy, affecting up to 40% of the global population (1). Significant economic and quality of life impacts are associated with AR including decreased productivity, cognitive function, and sleep (2, 3). AR is a heterogeneous disorder defined by IgE-mediated inflammation of the nasal mucosa. Allergic responses are initiated by environmental allergen exposure in genetically predisposed individuals, resulting in symptoms of rhinorrhea, sneezing, nasal congestion, and exacerbation of comorbid asthma (4). Children and adults with AR have an increased risk of developing allergic asthma (5, 6).

AR is characterized by an immediate early-phase response (EPR) and, in some individuals, a subsequent late-phase response (LPR). Allergen exposure in IgE-sensitized individuals causes degranulation of mast cells and basophils, inducing the EPR. The LPR occurs hours after allergen exposure and is caused by infiltration of inflammatory cells, including Th2 T lymphocytes, eosinophils, and basophils, into the nasal mucosa. Unlike the EPR, nasal congestion is the predominant symptom experienced during the LPR (7). Distinct immunological changes have been identified in the EPR (8, 9). However, there is limited understanding about the LPR, and how certain individuals may be protected from developing this additional allergic response. We have previously shown that polymorphisms in the cholinergic synapse pathway are associated with late asthmatic responses (10). Given the pathobiological overlap between allergic asthma and rhinitis, we hypothesized that this pathway might also be associated with AR sub-phenotypes.

In this investigation, we used the Environmental Exposure Unit (EEU) (11), a controlled allergen challenge facility, to study the development of the LPR. The EEU is a precise, replicable model of AR that offers the ability to study the development of allergic responses and symptoms, while removing confounding variables present in the natural environment. Using self-reported nasal congestion scores collected during EEU studies, allergic participants were sub-phenotyped either as acute congestion (AC) participants if they developed only an EPR, or as persistent congestion (PC) participants if they developed both early and late allergic responses. Genotyping and statistical analysis revealed an association between single nucleotide polymorphisms (SNP) in genes comprising the cholinergic synapse pathway and the LPR. This study also identified SNPs in *AKT3*, a cholinergic synapse pathway gene, which were associated with gene expression differences in PC participants.

## Methods

### Cohort

The institutional review boards of the University of British Columbia and Queen's University approved this study, and all participants provided written informed consent. In this investigation, 74 allergic and 20 healthy, non-allergic participants underwent allergen exposure. Allergic participants had a 2-year documented history of allergic rhinoconjunctivitis symptoms. Additionally, allergic participants had a positive skin prick test to either birch (*Betula pendula*), or rye grass (*Lolium perenne*), or house dust mite (*Dermatophagoides pteronyssinus* and *Dermatophagoides farinae*) at screening (defined as a wheal diameter of 3 mm or greater than that produced by the negative control) (12). Healthy participants had a negative skin prick test to common environmental allergens. Participants were of Caucasian ethnicity and between the ages of 18–65 years. Participants were excluded if they had an upper respiratory tract infection within 1 week of allergen exposure, asthma requiring the use of a short-acting beta-agonist greater than twice a week, and if they were unable to adhere to medication washout periods before screening and allergen exposure. Participants were also excluded if they had a known history of positive test results for Hepatitis B, Hepatitis C, HIV or tuberculosis, a significant history of alcohol or drug abuse, or clinically relevant abnormalities on physical exam.

### Environmental Exposure Unit

Using the EEU, participants were exposed to either birch pollen (3,500 grains/m^3^ for 4 h), or rye grass pollen (2,500–3,500 grains/m^3^ for 3 h), or house dust mite (2.07–6.66 ng/m^3^ of *D. pteronyssinus* and 2.67–3.80 ng/m^3^ of *D. farina* for 3 h). The air in the EEU was continuously circulated using fans, and allergen levels were measured every 30 min using impact-type particle samplers. The allergen emission rate was modified based on these counts to maintain target allergen concentrations. Participants self-reported nasal congestion scores (along with other AR symptoms) at baseline, every 30 min in the EEU, and every hour after leaving the EEU until 12 h had passed since allergen onset. AR symptoms were scored using a four-point scale (0–3). The scores were defined as the following: 0 = absence of symptoms, 1 = mild symptoms, 2 = moderate symptoms, and 3 = severe and intolerable symptoms.

Participants measured their Peak Nasal Inspiratory Flow (PNIF), an objective measure of nasal patency, at the same time intervals as AR symptoms using the In-Check meter (Clement Clark International Ltd, Essex, UK). Variation in raw PNIF scores was minimized by calculating the percent change in PNIF from baseline.

### Nucleic Acid Extractions

Whole peripheral blood samples were collected from participants before allergen exposure using PAXgene Blood RNA tubes (PreAnalytiX, Hombrechtikon, Switzerland). First, intracellular RNA was extracted from 5 mL of each PAXgene tube using the PAXgene Blood miRNA kit (PreAnalytiX). Then, from the remaining PAXgene tube solution (5 mL), DNA was extracted using a modified protocol. The solution was centrifuged, the resulting nucleic acid pellets were washed, resuspended, and incubated in buffers and proteinase K from the PAXgene Blood miRNA kit (PreAnalytiX). After protein digestion, the cell lysate was homogenized, and DNA was extracted using the PAXgene Blood DNA kit (PreAnalytiX).

### Genotyping

DNA (500 ng) from allergic and healthy participants were interrogated using Axiom SNP arrays (Affymetrix, Santa Clara, USA). We used the Axiom Analysis Suite Software to perform quality control checks and export genotypes into PLINK format. Using PLINK, SNPs that were not in Hardy-Weinberg equilibrium and had minor allele frequency <10% were excluded from the dataset. Allele frequencies of 218 SNPs that were located <50,000 bp upstream of the transcription start site and downstream of the 3′ untranslated region of *ADCY3, AKT3, CACNA1S, CHRM3, CHRNB2, GNB1, GNG4*, and *KCNQ4* were analyzed using logistic regression. Sex was controlled for in this analysis and we considered a nominal *P* value of <0.10 to be significant. Next, minor allele content (MAC) of PC, AC, and healthy participants was calculated (total number of minor alleles divided by the total number of SNPs analyzed), and the mean MAC values of each subgroup were compared using Mann–Whitney *U*-tests. Linkage disequilibrium was calculated using the LDheatmap R-library.

### Gene Expression

Peripheral blood RNA samples (100 ng) from allergic and healthy participants were interrogated using a custom NanoString nCounter Elements assay (NanoString, Seattle, USA). The assay measures the relative expression of 166 genes and has previously been used to develop blood-based biomarker panels to predict late-asthmatic responses. Reporter and Capture probes of the 166 target genes were mixed with each RNA sample for hybridization at 67°C for 18 h. The hybridized samples were processed using the nCounter Prep Station with the High Sensitivity protocol and then quantified using the nCounter Digital Analyzer. Raw data was normalized using positive controls and housekeeping genes. A cis-expression quantitative trait loci (cis-eQTL) analysis was performed using the MatrixEQTL R-library, and we considered an FDR < 0.01 to be significant.

## Results

### Participants and Nasal Congestion Development

Our goal was to test if polymorphisms in the cholinergic synapse pathway were associated with underlying nasal congestion development. Participants recorded their nasal congestion and PNIF scores over a 12-h period following allergen onset. A strong correlation was observed between nasal congestion and percent PNIF change from baseline (Pearson correlation, *R*^2^ = 0.96).

**Figure 1:**
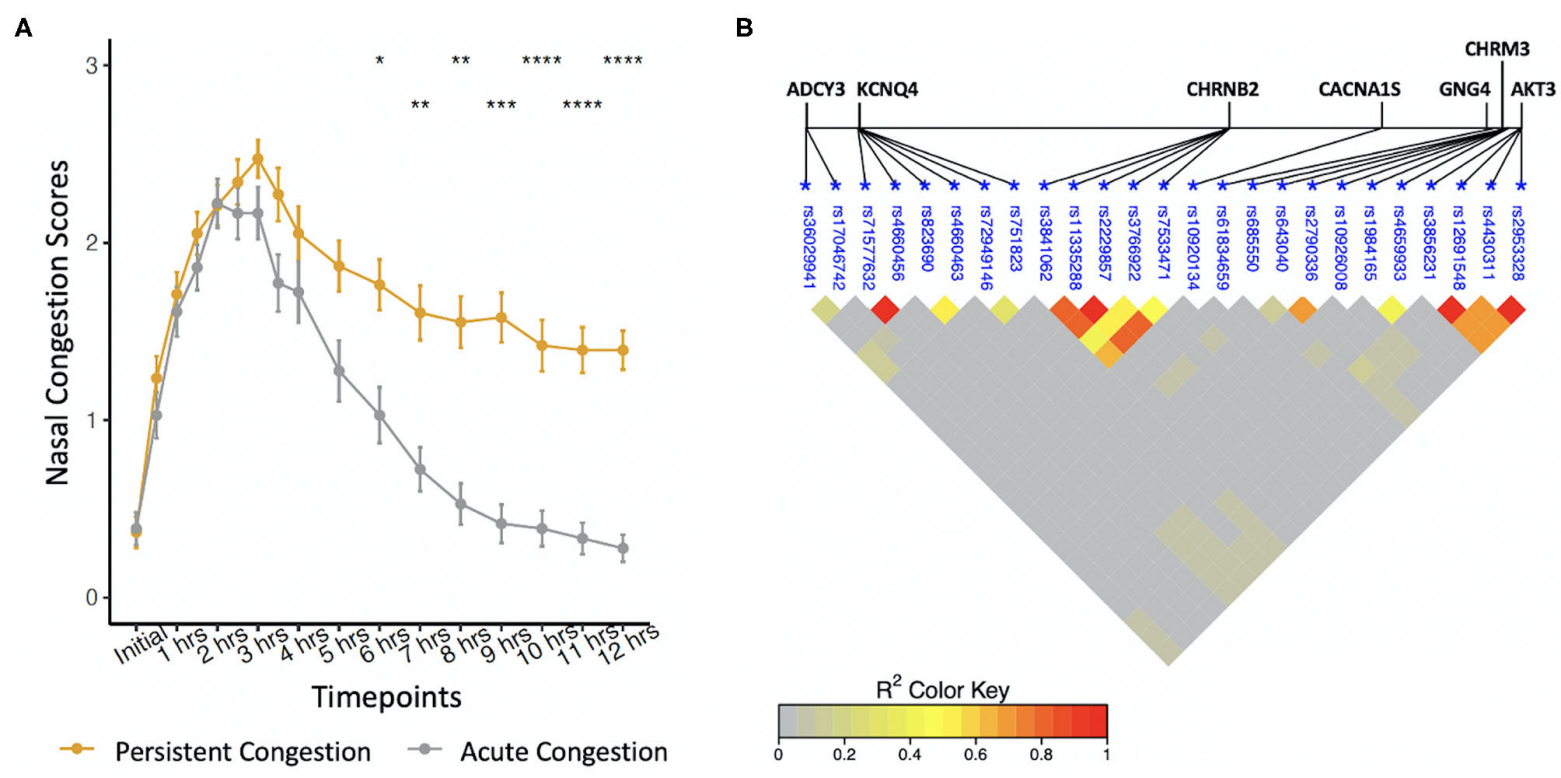
**(A)** Phenotyping of self-reported nasal congestion scores over a 12-h period from the start of allergen exposure. Significant differences were found between the sub-phenotypes at hours 6–12. *P < 0.1, **P < 0.01, ***P < 0.001, ****P < 0.0001, Wilcoxon Signed Rank test with Bonferroni corrections. **(B)** Linkage disequilibrium between polymorphisms associated with the late-phase response.

Allergic participants were sub-phenotyped using nasal congestion scores (Figure 1A). AC participants developed only an EPR; they experienced a 50% decrease in congestion scores between hours 3–6 and returned to baseline by hour 12. PC participants developed both allergic responses; they did not experience a 50% decrease in nasal congestion scores by hour 6 compared to hour 3 and did not return to baseline by hour 12. Of the 74 allergic participants as part of this investigation, 38 participants had PC and 36 participants had AC. During the first 5 h of allergen exposure, there were no significant differences among the sub-phenotypes (Wilcoxon Signed Rank test with Bonferroni corrections). Significant differences were seen at 6 h (*P* < 0.1), 7 and 8 h (*P* < 0.01), 9 h (*P* < 0.001), and from 10 to 12 h (*P* < 0.0001). Factors such as age, sex, height, weight, and complete blood cell counts did not differ between PC, AC, and healthy participants (Table 1).

**Table 1:** Demographics of research participants.

	**NA (*n* = 20)**	**PC (*n* = 38)**	**AC (*n* = 36)**	***P*-Value (NA vs. PC)**	***P*-Value (NA vs. AC)**	***P*-Value (PC vs. AC)**
Sex	14 F/6 M	20 F/18 M	22 F/14 M			
Height, cm^*^	166.83 ± 6.48	170.99 ± 9.44	168.09 ± 9.17	0.05	0.55	0.19
Weight, kg^†^	85.30 (75.70–101.68)	85.45 (72.55–96.45)	85.75 (68.48–91.45)	0.61	0.56	0.83
Age, yr^†^	38.50 (25.75–48.00)	39.00 (31.00–44.75)	40.00 (30.00–45.75)	0.94	0.70	0.67
Allergen						
Birch	9	14	15			
Grass	8	16	14			
HDM	3	8	7			
Blood cell counts and frequencies before challenge						
Leukocytes, ×10^9^ cells/L^*^	6.09 ± 0.84	6.49 ± 1.61	6.17 ± 1.65	0.22	0.82	0.39
Neutrophils, %^*^	59 ± 7	57 ± 7	56 ± 8	0.53	0.17	0.35
Lymphocytes, %^*^	29 ± 7	31 ± 6	32 ± 7	0.56	0.27	0.50
Monocytes, %^*^	7 ± 2	7 ± 2	8 ± 2	0.41	0.10	0.27
Eosinophils, %^†^	2 (2–4)	3 (2–3)	3 (2–4)	0.60	0.99	0.91

* *Variable is assumed to be normally distributed. Descriptive statistics are presented as mean SD. A t test was used to compare between the two groups*.

† *Variable is assumed to not be normally distributed. Descriptive statistics are presented as median (25–75th percentiles). A Wilcoxon rank-sum test was used to compare between the two groups. NA, non-allergic; PC, persistent congestion; AC, acute congestion; HDM, house dust mite*.

### Polymorphisms Associated With the Late-Phase Response

Allele frequencies of 25 SNPs were significantly different between PC and AC sub-phenotypes (Table 2). Identified SNPs were in seven genes comprising the cholinergic synapse pathway: *ADCY3, AKT3, CACNA1S, CHRM3, CHRNB2, GNG4*, and *KCNQ4*. Several of the SNPs were in linkage disequilibrium (Figure 1B). The average MAC of the PC sub-phenotype (MAC = 0.71) was significantly higher than that of the AC sub-phenotype (MAC = 0.61, *P* = 0.009). The 25 SNPs were not significantly different between healthy participants and either PC or AC participants.

**Table 2:** Significantly different SNPs between participants with persistent and acute congestion.

**Gene**	**RS number**	**Chromosomal location**	**Biological position**	**Minor allele**	**Odds ratios**	***P*-Value**
*ADCY3*	rs17046742	2	24942956	A	2.766	0.08432
	rs36029941	2	24904932	T	1.824	0.08664
*AKT3*	rs12691548	1	243656826	A	2.551	0.02473
	rs3856231	1	243605604	T	2.369	0.03715
	rs4430311	1	243852691	C	1.961	0.09573
	rs2953328	1	243860378	C	1.961	0.09573
*CACNA1S*	rs10920134	1	201148684	C	2.434	0.05842
*CHRM3*	rs1984165	1	239954965	T	0.4314	0.0262
	rs4659933	1	239955647	A	2.268	0.0271
	rs10926008	1	239898823	G	2.359	0.03645
	rs643040	1	239784120	C	0.5105	0.05141
	rs685550	1	239761108	G	0.427	0.05733
	rs2790336	1	239799386	G	0.5727	0.0891
*CHRNB2*	rs3766922	1	154604579	G	0.3891	0.008479
	rs11335288	1	154591260	G	2.112	0.03226
	rs2229857	1	154601491	T	2.112	0.03226
	rs7533471	1	154628860	G	1.886	0.05297
	rs3841062	1	154578468	–	1.877	0.07905
*GNG4*	rs61834659	1	235673662	T	4.529	0.04533
*KCNQ4*	rs71577632	1	40773241	TGGAG	0.2785	0.01629
	rs4660456	1	40773839	G	0.2785	0.01629
	rs751823	1	40875748	T	2.536	0.01666
	rs4660463	1	40799954	T	0.4024	0.02658
	rs823690	1	40796214	G	1.958	0.05236
	rs72949146	1	40860761	C	3.118	0.05742

*Odds ratio: for minor allele, major allele was used as reference*.

### Genetic Variants Associated With Gene Expression Differences in the Late-Phase Response

**Figure 2:**
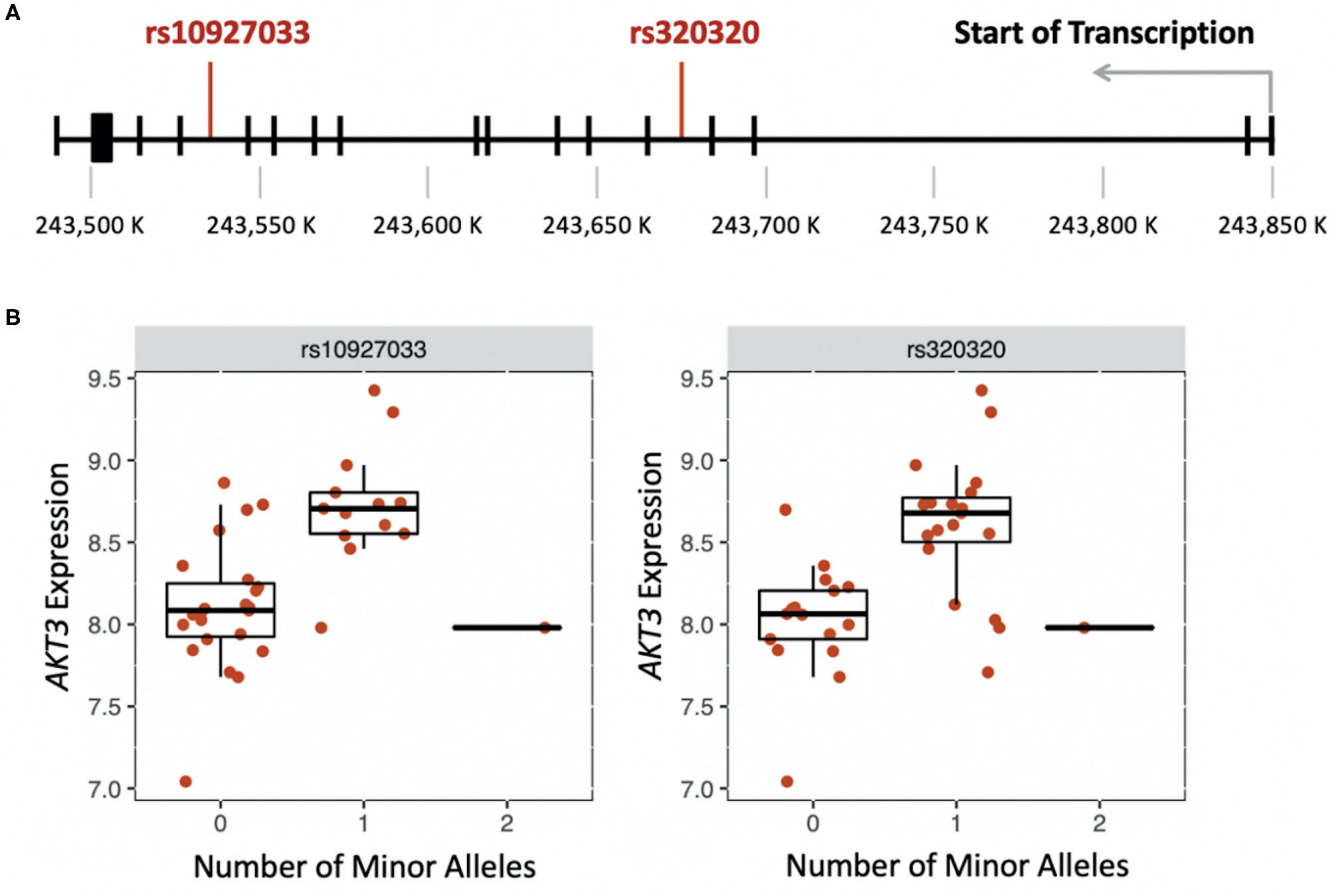
**(A)** Location of genetic variants that influence expression of *AKT3* in the late-phase response (FDR < 0.01). **(B)** Boxplots showing the effect of the number of minor alleles on *AKT3* expression for each genetic variant.

We were able to measure the relative RNA abundance (gene expression) for one of the seven cholinergic synapse pathway genes, *AKT3*. Thus, we performed a cis-eQTL analysis to identify genetic variants in *AKT3* associated with gene expression differences in PC, AC, and healthy participants. Using an additive and dominant genetic model, we identified two cis-eQTLs associated with the PC sub-phenotype (Figure 2A). The cis-eQTLs were in moderate linkage disequilibrium (*r* = 0.6). PC participants who were heterozygous for rs10927033 and rs320320 had significantly increased *AKT3* expression (Figure 2B).

## Discussion

Stimulation of parasympathetic cholinergic nerves results in tissue remodeling, reduced airflow, and hypersecretion. In this investigation, we were interested in investigating the cholinergic synapse pathway in AR. We studied nasal congestion during and after allergen exposure in a controlled setting, using the Environmental Exposure Unit (EEU), and developed a protocol to sub-phenotype AR. We identified an association between the late-phase response (LPR) and polymorphisms in cholinergic synapse pathway genes (*ADCY3, AKT3, CACNA1S, CHRM3, CHRNB2, GNG4*, and *KCNQ4)*. Persistent congestion (PC) participants who developed the LPR had a significantly increased minor allele content (MAC) than acute congestion (AC) participants who only developed the early-phase response (EPR).

Acetylcholine mediates cholinergic synapse transmission and is the predominant parasympathetic neurotransmitter in the airways. In the nose, acetylcholine causes nasal discharge and congestion (13). Increased activity of choline acetyltransferase, an enzyme that catalyzes acetylcholine biosynthesis, is associated with AR (14). This increased activity suggests that elevated neurotransmitter levels are present in individuals with AR, contributing to the development of rhinorrhea and nasal congestion symptoms after allergen exposure. *CHRM3* encodes a muscarinic receptor (M3) and *CHRNB2* encodes a subunit of nicotinic receptors, and both receptors bind acetylcholine. Muscarinic receptors are a class of G-protein-coupled receptor subtypes; M3 is the dominant muscarinic receptor in the nose and mediates secretion and vasodilation (14). Nicotinic receptors mediate the influx of cations using voltage-gated calcium (CACNA1S), potassium (KCNQ4), and sodium channels.

*ADCY3, AKT3*, and *GNG4* encode genes involved in G-protein coupled receptor signaling and mediate multiple signaling pathways. The *AKT3* gene is also involved in platelet activation (15), essential for leukocyte recruitment during allergen-induced inflammatory responses (16, 17). In addition to cytokines and chemokines, platelets sustain nasal inflammation by inducing an influx of inflammatory cells toward the nasal mucosa, a characteristic of the LPR (18). In AR, eosinophils adhere to cholinergic nerves, leading to their activation and degranulation. This interaction induces the expression of several cholinergic genes, such as choline acetyltransferase (19), resulting in elevated levels of acetylcholine and increased nasal symptoms. We also identified genetic variants in *AKT3* associated with gene expression differences in the blood of PC participants. This association was not observed in AC and healthy participants. To our knowledge, these cis-eQTLs have previously not been associated with AR or other conditions.

This investigation has several limitations. Although participants' scoring of nasal congestion strongly correlated with percent PNIF change from baseline, nasal congestion scores are a subjective measure. Despite this limitation, allergic participants were sub-phenotyped using self-reported nasal congestion scores, in accordance with the FDA guidance on clinical outcomes for AR studies (20). Additionally, previous studies have also used self-reported symptom scores to stratify AR participants (21, 22). Another limitation was combining allergic and healthy participants from several different EEU studies, which used either seasonal or perennial allergens. However, for each study, allergen concentrations and exposure times were previously determined and proven to generate a mean peak of six for total nasal symptom scores (TNSS; a composite score of sneezing, rhinorrhea, and nasal congestion and itching) (23–25). Participants achieving a TNSS of 6 is a standard for controlled allergen challenge facility studies (26). Future studies should investigate the cholinergic synapse pathway separately in seasonal and perennial AR. This study also had a relatively small sample size which limited our statistical power. We investigated the cholinergic synapse pathway in AR sub-phenotypes because it has been associated with the LPR in allergic asthma (10). Our results are consistent with the findings in asthma, and the sample sizes of these two studies are similar. A strength of this investigation was the inclusion of healthy individuals, which helped to ensure that the identified polymorphisms were specific to AR and the LPR.

We have shown for the first time that polymorphisms in cholinergic synapse pathway genes are associated with the LPR in AR. We have also demonstrated that nasal congestion scores are a useful measurement to sub-phenotype the LPR. Anticholinergic therapy in AR reduces the duration of rhinorrhea (27, 28), and our results suggest that similar treatments may also reduce the duration of nasal congestion.

## Data Availability Statement

The data analyzed in this study is subject to the following licenses/restrictions: Datasets relevant to the results presented in this paper will be made available through direct request to the corresponding author. Requests to access these datasets should be directed to Scott Tebbutt, scott.tebbutt@hli.ubc.ca.

## Ethics Statement

The studies involving human participants were reviewed and approved by the University of British Columbia and Queen's University. The patients/participants provided their written informed consent to participate in this study.

## Author Contributions

SS, AR, AE, and ST designed the study. SS and AE performed experiments. SS, AS, and ST participated in the statistical analysis. SS wrote the first draft of the manuscript. All authors contributed to the final version of the manuscript.

## Conflict of Interest

The authors declare that the research was conducted in the absence of any commercial or financial relationships that could be construed as a potential conflict of interest.

## Publisher's Note

All claims expressed in this article are solely those of the authors and do not necessarily represent those of their affiliated organizations, or those of the publisher, the editors and the reviewers. Any product that may be evaluated in this article, or claim that may be made by its manufacturer, is not guaranteed or endorsed by the publisher.
